# Effect of Gd Alloying on Magnetic Properties of Direct-Quenched Fe-Gd-B Nanocrystalline Alloys

**DOI:** 10.3390/ma19030561

**Published:** 2026-01-30

**Authors:** Linli Wang, Yuanyuan Wang, Zhongao Wang, Ming Nie, Feng Huang, Wangyan Lv, Huameng Fu, Haifeng Zhang, Zhengwang Zhu

**Affiliations:** 1 Electric Power Research Institute of Guangdong Power Grid Co., Ltd., Guangzhou 510080, China; ll_wang25@163.com (L.W.); zh_wang2018@163.com (Z.W.); ming_pro1@163.com (M.N.); huanfenhf@163.com (F.H.); yan_tech2011@163.com (W.L.); 2 Institute of Metal Research, Chinese Academy of Sciences, Shenyang 110016, China; wangyuanyuan@smm.neu.edu.cn (Y.W.); hmfu@imr.ac.cn (H.F.); zhanghf@mail.neu.edu.cn (H.Z.)

**Keywords:** Fe-Gd-B alloys, nanocrystalline materials, magnetic properties, microstructure, domain wall pinning

## Abstract

Nanocrystalline Fe-Gd-B alloys were successfully synthesized via Gd alloying in a binary Fe-B system using a single-roller melt-spinning technique. A systematic investigation of Gd content variation (0–4.35 at.%) reveals its critical role in tuning microstructure evolution, thermal stability, and magnetic properties. Crucially, the Fe_90.70_Gd_2.32_B_6.98_ alloy ribbon exhibits optimized magnetic performance, achieving a high saturation magnetic induction (*B_s_*) of 1.67 T and a low coercivity (*H*_c_) of 2.737 kA/m. This enhancement is attributed to the suppression α-Fe grain growth through Gd-induced elevation of the thermal stability of the amorphous matrix, which confines the average crystallite size to 26.3 nm. The refined α-Fe phase contributes to elevated *B_s_* through an increased ferromagnetic fraction, while its nanoscale grain structure, combined with wide magnetic domain configurations, effectively reduces *H*_c_ by limiting domain wall pinning sites. These findings establish that the synergistic effect of Gd alloying and Fe/B ratio adjustment is a viable strategy for designing high-performance Fe-based magnetic alloys.

## 1. Introduction

The pursuit of enhanced magnetic properties in Fe-based materials has persisted since the discovery of electromagnetic pure iron over a century ago, as they are critical for power electronics, high-frequency transformers, and advanced magnetic devices. To date, reported magnetic materials primarily include pure iron, silicon steel, ferrites, and amorphous/nanocrystalline alloys [1,2,3,4,5]. Among these, Fe-based nanocrystalline alloys have attracted extensive attention due to their excellent properties. Compared with Fe-based amorphous alloys, Fe-based nanocrystalline alloys exhibit lower coercivity (*H*_c_), thereby enhancing electromagnetic conversion efficiency and reducing hysteresis loss [6,7]. Previous studies have shown that the Fe-B binary alloys exhibit a strong amorphous forming ability compared to other binary alloys containing Fe due to the existence of deep eutectic point [8,9]. Yoshizawa et al. discovered that adding Cu and Nb elements combined with appropriate annealing could significantly improve the soft magnetic properties of Fe-Si-B alloys, leading to the development of the representative FINEMET alloy [10]. Subsequently, Fe-Zr-B, Fe-Hf-B, and Fe-M-B-Cu (M = Ti, Zr, Hf, Nb, or Ta) alloy systems were successively developed and collectively referred to as NANOPERM alloys [11]. Furthermore, (Fe, Co)-M-B-Cu (M = Nb, Hf, or Zr) nanocrystalline alloys were developed and trademarked as HITPERM [12]. At present, these various Fe-based nanocrystalline alloys have been widely applied in electronic components [13,14]. However, these alloys either suffer from complex preparation processes and high production costs due to their high content of rare metals, or exhibit low saturation magnetic induction resulting from high content of non-magnetic elements, thereby limiting their application scope.

Typically, the preparation of Fe-based nanocrystalline alloys requires obtaining an amorphous precursor at first, followed by heat treatment to promote the precipitation of nanoscale α-Fe grains, forming a nanocrystalline–amorphous dual-phase structure [15]. The precipitation of nanocrystals plays a crucial role in soft magnetic properties. It is generally recognized that Cu is widely used to induce uniform nanocrystal precipitation [12,13,14], primarily due to the positive mixing enthalpy between Cu and Fe [16,17]. During rapid cooling, Cu forms extremely small and uniformly distributed clusters, which serve as heterogeneous nucleation sites during subsequent heat treatment, facilitating the precipitation of α-Fe phase and resulting in a uniformly distributed nanocrystalline structure [18,19]. However, for nanocrystalline ribbons, excessively long annealing times can reduce free volume, leading to significant embrittlement [20]. To address this issue, some strategies such as ultra-rapid annealing or low-temperature processing have been explored to achieve nanocrystalline–amorphous composite structures [21], demonstrating improved thermal stability through grain boundary relaxation mechanisms. In recent years, a novel preparation strategy has been proposed to simplify the fabrication of nanocrystalline magnetic materials [22]. This approach enables direct formation of nanocrystalline phases during melt-spinning via rapid cooling on a rotating copper roller, followed by low-temperature, short-duration annealing to relieve internal stresses and enhance soft magnetic properties. The method not only effectively suppresses ribbon brittleness but also streamlines the manufacturing process while reducing energy consumption [22]. Nevertheless, the limited amorphous forming ability has restricted the direct formation of nanocrystalline–amorphous dual-phase structure via melt-spinning.

To address this challenge, a novel alloy composition was designed by increasing Fe content and incorporating Gd within the Fe-B binary system in the present work. It is important to note that in practical alloy design, the introduction of Gd is rarely an isolated variable. In this study, to maintain glass-forming ability (GFA) and balance the phase volume fractions, the addition of Gd was accompanied by concurrent adjustments to the Fe and B content. Unlike previous studies, this study systematically investigates the synergistic effects of Gd minor-alloying and the consequential Fe/B ratio variation on the microstructure, thermal stability, and magnetic properties of Fe-Gd-B alloys. By correlating the evolution of phase composition, grain size and magnetic domain structures with the observed magnetic properties, we aim to provide a comprehensive understanding of how Gd alloying tailors the magnetic behavior of Fe-based nanocrystalline alloys.

## 2. Experimental

All ingots with nominal compositions of Fe_87_B_13_, Fe_90.47_Gd_1.59_B_7.94_, Fe_90.56_Gd_1.89_B_7.55_, Fe_90.70_Gd_2.32_B_6.98_, Fe_90.91_Gd_3.03_B_6.06_ and Fe_91.30_Gd_4.35_B_4.35_ (in atomic percentage, denoted as Gd0, Gd159, Gd189, Gd232, Gd303 and Gd435, respectively, in the following) were prepared by arc melting under an argon atmosphere. The raw materials used in this work include industrial Fe-B alloy (Fe_83_B_17_, 99.9 wt%), pure Fe (99.9 wt%), and pure Gd (99.9 wt%). Alloy ribbons with a thickness of 22 µm and a width of 0.8 mm were fabricated using single-roller melt spinning with a surface linear velocity of 50 m/s under an argon protective atmosphere. The as-spun ribbons were sealed in vacuumed quartz tubes and subsequently isothermally annealed in a muffle furnace at the temperatures of 300–550 °C for 30 min, followed by water quenching to relieve internal stress. The heating rate to the set annealing temperature is around 20 K/min. The microstructure of the as-spun and annealed alloy ribbons was analyzed by X-ray diffraction (XRD, Rigaku MiniFlex, Rigaku Corporation, Tokyo, Japan) with Cu K*_α_* radiation (λ = 0.154056 nm) and transmission electron microscopy (TEM, ThemoFisher Talos F200x, Thermo Fisher Scientific, Waltham, MA, USA). Thermal properties of the as-spun ribbons, including the primary crystallization temperature (*T*_x1_) and secondary crystallization temperature (*T*_x2_), etc., were determined by differential scanning calorimetry (DSC, Simultaneous Thermal Analyzer 8000, PerkinElmer, Hopkinton, MA, USA) at a heating rate of 0.67 K/s. The density of the samples was measured using the Archimedes drainage method in distilled water, with a measurement accuracy of 0.01 g·cm^−3^. The saturation magnetic flux density (*B*_s_) of the alloy ribbons were measured using a vibrating sample magnetometer (VSM, East Changing VSM-3105, East Changing, Shanghai, China) under a magnetic field of 160 tkA·m^−1^ paralleled to the freeside of the ribbons, and the coercivity (*H*_c_) of the alloy ribbons were measured using a DC *B-H* loop tracer (Linkjoin MATS-2010SD, Lianjie Technology, Beijing, China). Additionally, the magnetic domain structure of the ribbons was observed using atomic force microscopy (AFM, HR-AFM Grapes-Tech, Grapes Tech, Shenzhen, China).

## 3. Results and Discussion

**Figure 1:**
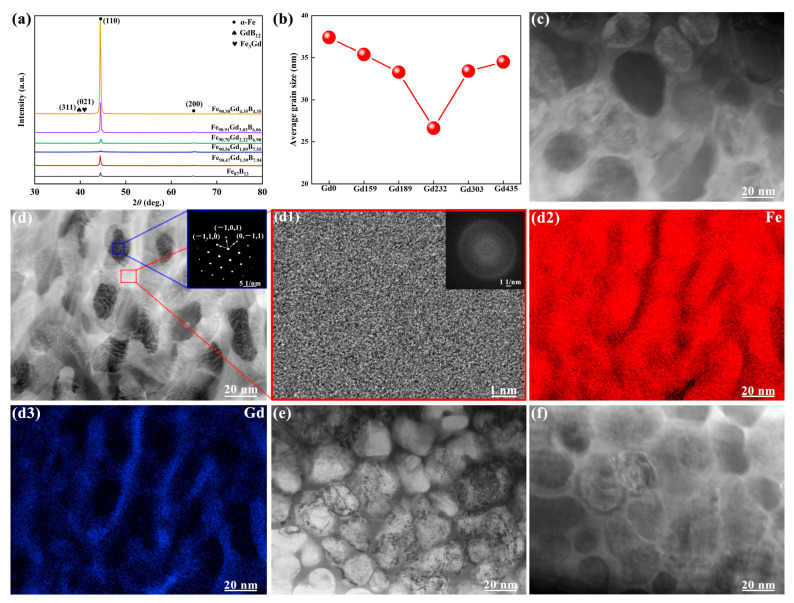
(**a**) XRD patterns and (**b**) average α-Fe grain sizes of the as-spun Fe-Gd-B ribbons. (**c**) Bright-field TEM image of the Gd159 alloy. (**d**) Bright-field TEM image of the Gd232 alloy (inset: SAED pattern). (**d1**) HRTEM image of the red boxed area in (**d**) showing nanocrystalline structure (inset: FFT pattern). (**d2**,**d3**) EDS elemental maps showing the distribution of Fe and Gd, respectively. (**e**,**f**) Bright-field TEM image of the Gd303 and Gd435 alloy.

The structures of all alloy samples were characterized as shown in Figure 1. The Fe_87_B_13_, Fe_90.47_Gd_1.59_B_7.94_, Fe_90.56_Gd_1.89_B_7.55_, Fe_90.70_Gd_2.32_B_6.98_, Fe_90.91_Gd_3.03_B_6.06_ and Fe_91.30_Gd_4.35_B_4.35_ ribbons displayed distinct crystalline diffraction peaks at approximately 45°and 65°, respectively. These diffraction peaks were identified as the (110) and (200) crystal planes of the α-Fe phase, respectively, through comparative analysis with standard PDF cards. As the Gd content increases, the intensity of the (110) peak firstly increases and then decreases, indicating that Gd promotes α-Fe precipitation. However, as the Fe content decreases, the effect of Gd on promoting α-Fe precipitation weakens. It is obviously seen that these diffraction peaks exhibit significant broadening, which can be primarily attributed to the grain refinement effect. The grain size was calculated based on the XRD diffraction peaks using the Scherrer formula, as follows:

D = (Kλ)/(βcos θ),

where D, K, λ, β and θ corresponding to average crystalline size in the diffraction direction, Scherrer constant, X-ray wavelength, full width at half maximum (FWHM) of the diffraction peak and Bragg diffraction angel, respectively. The grain size was in the range of 26~38 nm for the precipitated α-Fe phases in the current alloys, as shown Figure 1b. The Gd232 alloy shows the smallest average size for the precipitated α-Fe phases among all the current alloys.

To validate this, the microstructure of the Gd159, Gd232, Gd303 and Gd435 alloy was further examined using TEM, Thermo Fisher Scientific, Waltham, MA, USA. The bright-field image in Figure 1c–f reveals a uniform nanocrystalline morphology, where α-Fe grains with diameters of 25–30 nm are homogeneously embedded within a residual amorphous matrix. This direct observation aligns perfectly with the XRD calculations. The corresponding selected area electron diffraction (SAED) pattern was inset with Figure 1d, displaying BCC structure. Crucially, the high-resolution TEM (HRTEM) image of the intergranular region, shown in Figure 1(d1), exhibits a disordered atomic arrangement lacking long-range periodicity. The corresponding Fast Fourier Transform (FFT) pattern (inset of Figure 1(d1)) displays a typical diffuse halo, characteristic of an amorphous structure. This confirms that the precipitated α-Fe nanocrystals are embedded within a thin layer of residual amorphous matrix, forming a classic nanocrystalline/amorphous composite structure. Finally, the elemental distribution was analyzed via EDS mapping, as shown in Figure 1(d2,d3). The mapping results demonstrate that while Fe is enriched in the grains, Gd atoms (Figure 1(d3)) tend to be enriched at the grain boundaries or intergranular regions. This segregation behavior of the large-radius rare-earth atoms creates a solute drag effect, which restricts the diffusion of Fe atoms and effectively hinders the coarsening of α-Fe grains, thereby contributing to the refined microstructure observed in the Gd232 alloy.

**Figure 2:**
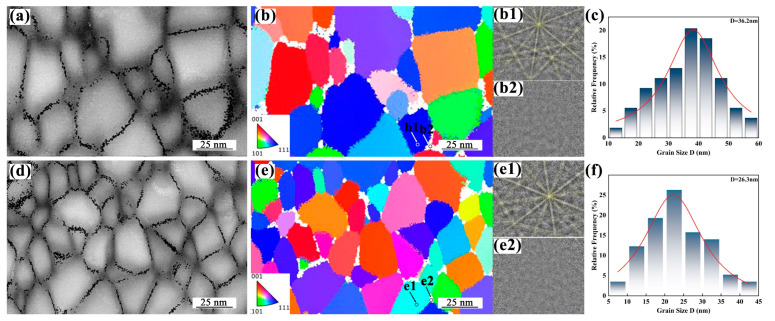
TKD characterization of Gd0 and Gd232 alloy ribbons: (**a**–**c**) Image quality map, phase distribution, diffraction patterns and grain size statistics for Gd0 alloy; (**b1**,**b2**)TKD patterns corre-sponding to pixels indicated by arrows in (**b**); (**d**–**f**) Corresponding datasets for Gd232 alloy; (**e1**,**e2**) TKD patterns corresponding to pixels indicated by arrows in (**e**).

**Figure 3:**
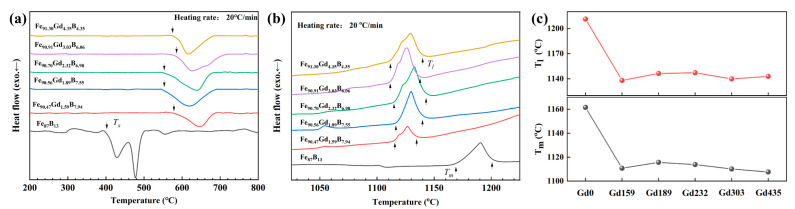
Thermal properties of melt-spun Fe-Gd-B alloy ribbons: (**a**) DSC curves of glass transition and crystallization events, (**b**) DSC curves of melting behavior, and (**c**) variation in solidus (T_m_) and liquidus (T_l_) temperatures with Gd content.

To further elucidate the microstructural characteristics of the as-spun Fe-Gd-B alloy ribbons, Electron Backscatter Diffraction with Transmission Kikuchi Diffraction (TKD) measurements were performed on the Gd0 and Gd232 alloys, as shown in Figure 2. The TKD phase maps reveal a distinct dual-phase microstructure consisting of grains indexed as the α-Fe phase (colored regions) and intergranular unindexed regions (dark regions). While unindexed areas in TKD can sometimes originate from surface defects or overlapping patterns, integrating the multi-technique characterization in this study supports the inference that these dark regions are predominantly the residual amorphous phase. This conclusion is corroborated by the XRD and TEM results (Figure 1), which exhibit amorphous halos, and the DSC curves (Figure 3), which show distinct exothermic crystallization peaks for all samples. For the Gd0 alloy, the Image Quality (IQ) map (Figure 2a) displays relatively coarse grains with well-defined boundaries. The corresponding Inverse Pole Figure (IPF) map (Figure 2b) visualizes the crystallographic orientations, displaying a diverse color palette (e.g., <001>, <101>, and <111>). This indicates that the Gd0 ribbon consists of a BCC phase with random orientation and no significant texture. The statistical analysis of grain size (Figure 2c) shows a distribution primarily in the range of 20–50 nm, with a peak at approximately 36.2 nm. In contrast, the Gd232 alloy exhibits a significantly refined microstructure. As shown in the phase map (Figure 2e), the alloy comprises α-Fe grains with random orientation, separated by boundary regions. The grain size statistics (Figure 2f) reveal that the grains in the Gd232 alloy are much finer, concentrated in the 15–35 nm range, with a narrower distribution peak at around 26.3 nm. Overall, the TKD characterization provides direct visual evidence that Gd alloying induces a significant microstructural refinement process, resulting in a smaller and more uniform grain structure compared to the Gd0 alloy. These results are in excellent agreement with the crystallite sizes calculated from XRD measurements. The formation of such fine and uniform grains in the directly solidified Gd232 alloy is a critical factor contributing to its enhanced magnetic properties.

To further interpret the effect of Gd alloying on the microstructure and thermal properties of Fe-Gd-B alloys, differential scanning calorimetry (DSC) was performed on all as-spun ribbons, as shown in Figure 3 and the specific values of the characteristic parameters are presented in Table 1. Figure 3a displays the DSC curves obtained at a heating rate of 20 °C/min within the temperature range of 200–800 °C. For the Gd0 alloy, the as-spun ribbons exhibit multi-stage crystallization behavior beginning at approximately 400 °C. In contrast, the Gd-containing alloys show a distinct, single-stage crystallization peak at a significantly higher temperature of about 600 °C. This remarkable upward shift of nearly 200 °C indicates that Gd addition substantially enhances the thermal stability of the residual amorphous phase. This stabilization is closely linked to the compositional evolution of the amorphous matrix. Since Gd is virtually insoluble in α-Fe (Figure 1e), the precipitation of α-Fe nanocrystals rejects Gd atoms into the remaining liquid, resulting in a Gd-enriched residual amorphous phase (Figure 1f). The high concentration of large Gd atoms exacerbates the atomic size mismatch with Fe, thereby increasing topological disorder and elevating the activation energy required for atomic rearrangement in the undercooled melt. Consequently, the residual amorphous matrix becomes highly resistant to further crystallization. As shown in Figure 3b,c, Gd alloying markedly alters the melting behavior. The introduction of Gd reduces the solidus and liquidus temperatures (T_m_ and T_l_, respectively) by approximately 60–70 °C. When combined with the significant elevation of the onset-crystallization temperatures (Figure 3a), the reduced glass transition temperature (T_rg_ = T_x_/T_l_) exhibits a marked increase with rising Gd content. This trend serves as a key thermodynamic indicator of enhanced glass-forming ability (GFA).

It seems contradictory due to enhanced GFA coupled with nanocrystallization in the current Gd-containing alloys, which can be rationalized by the unique modulation of Gd on solidification kinetics. On the one hand, the addition of Gd is hypothesized to induce compositional fluctuations or metastable phase separation in the undercooled melt. This heterogeneity generates abundant Fe-rich clusters that act as precursors for α-Fe nucleation, thereby significantly increasing the nucleation site density. On the other hand, the subsequent growth of these nuclei is severely restricted. As Gd atoms are rejected from the crystallizing α-Fe, they segregate at the growth interface, creating a strong effect of solute drag. This distinctive structure is pivotal for achieving superior magnetic properties. It is particularly noteworthy that in the current work, the addition of the Gd element was accompanied by the variations in the Fe/B ratio. Consequently, the observed changes in melting behavior and crystallization temperatures reported herein arise from the synergistic effects of Gd alloying and the Fe/B ratio.

**Figure 4:**
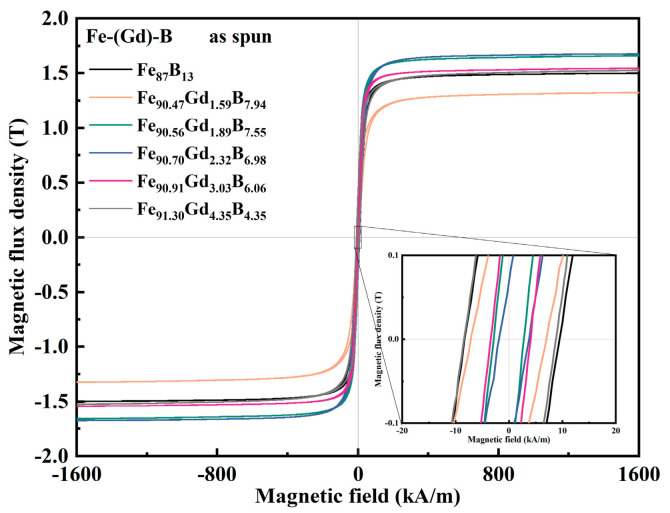
Hysteresis Loops of Melt-spun Fe-Gd-B System Alloy Ribbons.

**Figure 5:**
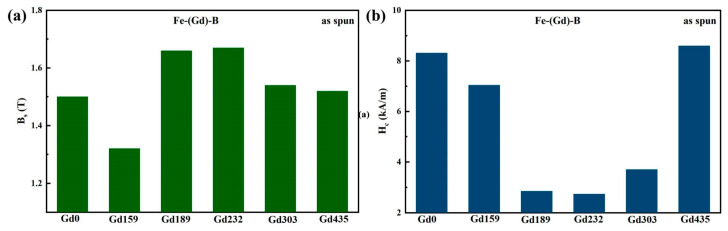
Soft magnetic properties of melt-spun Fe-Gd-B alloy ribbons: (**a**) saturation magnetic induction, and (**b**) coercivity.

**Figure 6:**
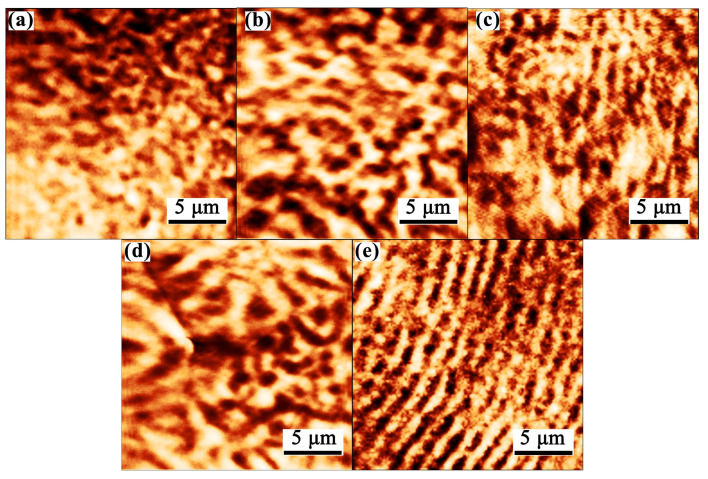
Magnetic Domain Structure of Melt-spun Fe-Gd-B System Alloy Ribbons: (**a**) Gd159, (**b**) Gd189, (**c**) Gd232, (**d**) Gd303 and (**e**) Gd435.

The magnetic properties of the as-spun Fe-Gd-B ribbons were evaluated using a vibrating sample magnetometer (VSM). Figure 4 displays the hysteresis loops for all as-spun samples. The key magnetic parameters, saturation magnetic induction (*B_s_*) and coercivity (*H_c_*), are summarized as a function of Gd content in Figure 5. As shown in Figure 5a, *B_s_* exhibits a non-monotonic evolution with Gd addition. It initially decreases but subsequently reaches a distinct peak of 1.67 T for the Gd232 alloy. This maximum is attributed to the optimized microstructure, where the high volume fraction of precipitated ferromagnetic α-Fe nanocrystals effectively compensates for the dilution effect and the antiferromagnetic coupling between Gd and Fe atoms. As presented in Figure 5b, *H_c_* follows a sensitive U-shaped dependence on composition, reaching a minimum value of 2.737 kA/m for the Gd232 alloy. It is noted that the overall coercivity levels for these as-spun ribbons are relatively high. This characteristic is primarily attributed to the substantial internal stresses induced by the rapid solidification process and the inherent local magnetocrystalline anisotropy introduced by the rare-earth Gd atoms. However, the relative reduction in *H_c_* at the optimal composition is significant. This improvement is consistent with Herzer’s Random Anisotropy Model (RAM). For the optimized Gd232 alloy, the refined grain size of ~26.3 nm is smaller than the ferromagnetic exchange coupling length (~35 nm). Consequently, the local magnetocrystalline anisotropies are effectively averaged out by the exchange interaction, leading to the observed minimum in coercivity. For the binary Gd0 or high-Gd samples, the higher *H_c_* suggests a breakdown of this averaging effect, likely due to larger grain sizes or increased pinning from structural heterogeneity, consistent with the fragmented domain structures observed in Figure 6.

To elucidate the micromagnetic mechanism, the magnetic domain structures were investigated via AFM. Figure 6 illustrates the magnetic domain configurations of the as-spun Fe-Gd-B ribbons, revealing a strong correlation between domain morphology and the U-shaped coercivity trend. The optimized Gd232 alloy (Figure 6c) specifically exhibits considerably wide and continuous strip-like domain structures. The observation of such large-scale magnetic domains suggests a reduced density of pinning sites (e.g., grain boundaries, defects) within the alloy, implying that domain walls experience less resistance and can move more freely upon the application of a small magnetic field. This facilitated domain wall motion is the primary reason for the minimum coercivity (*H_c_*) observed in this sample. Although XRD confirms that the alloy consists of nanoscale α-Fe grains, these large-scale domains indicate that intergranular exchange coupling interactions remain sufficiently strong to maintain coherent magnetization directions across multiple grains, effectively averaging out the local magnetocrystalline anisotropy. Conversely, the low-Gd samples (Figure 6a,b) display irregular patchy patterns, and the high-Gd sample (Figure 6e) exhibits extremely dense, fine stripes. As theoretically predicted, such finer or fragmented domain structures indicate stronger pinning effects and higher local anisotropy, which correspond directly to the increased *H_c_* values measured in these compositions. The formation of these domains arises from the system’s tendency to minimize demagnetizing field energy [23,24], and the evolution from fine/irregular to wide domains confirms that through rational microstructural control (i.e., optimizing Gd content), the effective magnetic anisotropy can be minimized to improve magnetic softness [25].

**Figure 7:**
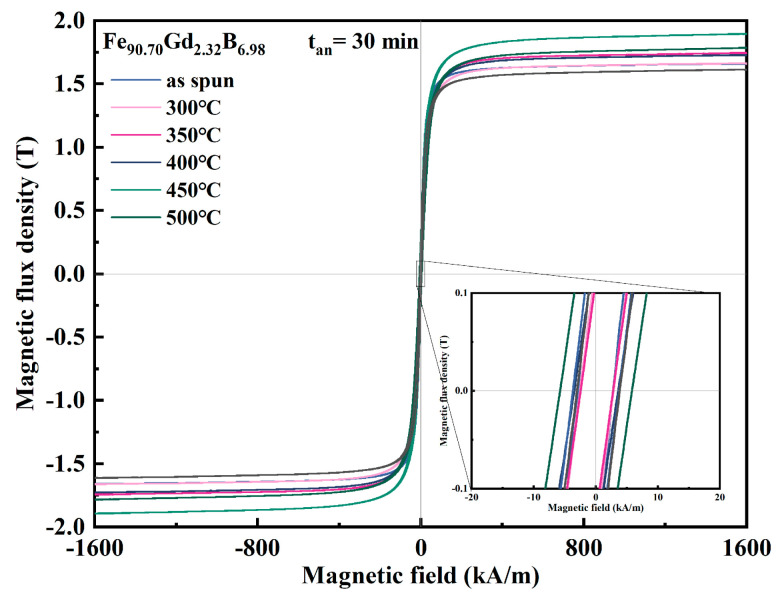
The hysteresis loop of the Fe_90.70_Gd_2.32_B_6.98_ alloy ribbons after annealing.

**Figure 8:**
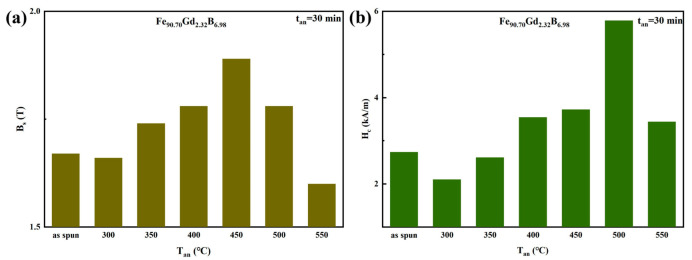
Soft magnetic properties of the Fe_90.70_Gd_2.32_B_6.98_ Ribbon after Annealing: (**a**) Saturation magnetic flux density *B_s_*; (**b**) Coercivity *H*_c_.

To further investigate the thermal stability and optimize the magnetic performance of the Fe-Gd-B nanocrystalline alloys, the Gd232 ribbon was annealed within the temperature range of 300–550 °C for 30 min (t_an_ = 30 min). Figure 7 presents the hysteresis loops of the alloy ribbon before and after heat treatment, while Figure 8 summarizes the corresponding evolution of B_s_ and *H_c_*. As shown in Figure 8a, the annealing temperature exerts a distinct influence on *B_s_*. Annealing at 300 °C resulted in a negligible change in *B_s_*, maintaining the level of the as-spun state. However, as the temperature increased to the range of 350–450 °C, *B_s_* exhibited a substantial increase, reaching a maximum of approximately 1.85 T at 450 °C. This enhancement is attributed to the thermally activated growth of α-Fe grains, which increases the volume fraction of the ferromagnetic crystalline phase relative to the residual amorphous matrix. Conversely, annealing at higher temperatures (500 °C and 550 °C) led to a sharp decline in B_s_. This deterioration is caused by the precipitation of non-magnetic or magnetically hard boride phases (e.g., Fe_2_B, Fe_3_B), which disrupt the ferromagnetic continuity.

The evolution of *H_c_*, shown in Figure 8b, reveals a sensitive dependence on the annealing stage, governed by the competition between stress relief and phase transformation. Firstly, when the annealing temperature is below 350 °C, the coercivity reached its minimum value after annealing at 300 °C. Since the as-spun ribbon is primarily composed of nanocrystals with a very low volume fraction of residual amorphous phase (Figure 1 and Figure 2), the dominant mechanism in this low-temperature regime is the release of internal quenching stresses. The rapid solidification process introduces significant macroscopic and microscopic stresses, which contribute to coercivity via magneto-elastic anisotropy. Annealing at 300 °C effectively relieves these stresses and relaxes the disordered structure at the grain boundaries, without inducing further grain growth or phase precipitation. This reduction in magneto-elastic anisotropy facilitates domain wall motion, resulting in the observed minimum in *H_c_*. Secondly, upon annealing at 350 °C and above, *H_c_* began to rise gradually [26,27]. This increase marks the onset of secondary crystallization or grain growth, where the coarsening of α-Fe grains weakens the intergranular exchange coupling effect [28]. The soft magnetic properties are primarily governed by the exchange coupling between nanocrystalline grains through the amorphous matrix. This coupling can be reinforced by increasing the saturation magnetization of the crystalline phases. The deterioration becomes most pronounced at 500 °C, where *H_c_* spikes drastically (exceeding 5.5 kA/m), as evidenced by the significantly widened hysteresis loop in Figure 7. This sharp hardening is probably attributed to the precipitation of hard magnetic Fe-B compounds, which act as strong pinning centers for domain wall motion. Although *H_c_* shows a decrease at 550 °C relative to the peak at 500 °C, the comprehensive magnetic property remains inferior to the optimized state. This systematic evolution of magnetic property confirms that heat treatment is a powerful tool for regulating the microstructure of Fe-Gd-B alloys. While 300 °C annealing yields the lowest coercivity through stress relaxation, 450 °C annealing maximizes the saturation induction. However, excessive temperatures (>500°C) trigger detrimental phase transformations that degrade both properties. Thus, a precise control of the annealing process is essential to achieve a balanced combination of high *B_s_* and low *H_c_* for specific application requirements.

**Figure 9:**
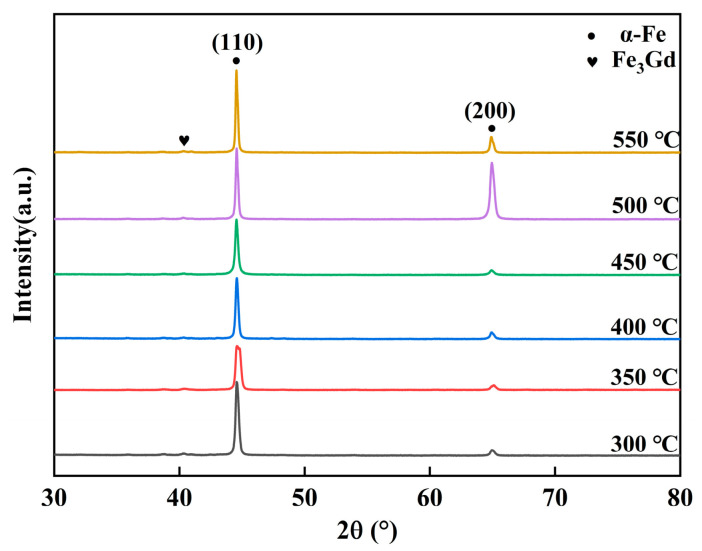
XRD patterns of Fe-Gd-B alloy ribbons.

Figure 9 displays the microstructural characteristics of the Gd232 alloy after annealing within the temperature range of 300–550 °C. Comparative analysis with the standard PDF card confirms that these diffraction peaks correspond to the (110) and (200) crystal planes of the α-Fe phase. As the annealing temperature increases, the intensity of the (110) peak enhances, indicating that the annealing temperature promotes the precipitation of the α-Fe phase and facilitates the enhancement of *Bs*.

## 4. Conclusions

In this study, Fe-Gd-B nanocrystalline alloys were successfully fabricated using a single-roller melt-spinning technique. The effects of Gd alloying, accompanied by concurrent variations in the Fe/B ratio, on the microstructure, thermal stability, and magnetic properties were systematically investigated. The main conclusions are drawn as follows:

(1) Gd minor-alloying facilitates the direct formation of a uniform nanocrystalline–amorphous structure during melt-spinning. The Fe_90.70_Gd_2.32_B_6.98_ alloy achieves an optimal average grain size of 26.3 nm, as verified by both XRD calculations and TEM observations.
(2) The thermal stability of the amorphous matrix is significantly enhanced (by ~200 °C) due to the synergistic effect of Gd alloying and Fe/B ratio modulation, which increases the topological disorder and slows down crystallization kinetics via the solute drag effect.
(3) The Fe_90.70_Gd_2.32_B_6.98_ ribbon exhibits the best comprehensive magnetic performance, with *B_s_* = 1.67 T and *H_c_* = 2.737 kA/m. The low coercivity is physically linked to wide, continuous magnetic domains, signifying a reduction in the density of pinning sites within the refined microstructure.
(4) Low-temperature annealing (300 °C) optimizes magnetic performance through internal stress release. High-temperature treatment (>500 °C) leads to catastrophic deterioration due to grain coarsening and the precipitation of hard magnetic boride phases.

In summary, this work demonstrates that tuning the Fe/B ratio in conjunction with rare-earth Gd alloying is an effective strategy for developing high-performance Fe-based magnetic materials for power electronic applications. Concurrently, future studies will involve the independent variation in Gd and B contents to decouple their individual contributions and further refine the alloy design criteria.

## Figures and Tables

**Table 1:** Thermodynamic parameters of Fe-Gd-B series alloys.

Alloy	*T*_x_ (°C)	*T*_m_ (°C)	*T*_l_ (°C)
Gd0	394	1162	1211
Gd159	555	1111	1138
Gd189	546	1116	1146
Gd232	545	1114	1147
Gd303	563	1110	1140
Gd435	571	1108	1143

## Data Availability

The original contributions presented in this study are included in the article. Further inquiries can be directed to the corresponding author.
